# Optimal Polygon Decomposition for UAV Survey Coverage Path Planning in Wind

**DOI:** 10.3390/s18072132

**Published:** 2018-07-03

**Authors:** Matthew Coombes, Tom Fletcher, Wen-Hua Chen, Cunjia Liu

**Affiliations:** Department of Automotive and Aeronautical Engineering, Loughborough University, Loughborough LE11 3TU, UK; T.P.Fletcher@lboro.ac.uk (T.F.); w.chen@lboro.ac.uk (W.-H.C.); C.Liu5@lboro.ac.uk (C.L.)

**Keywords:** dynamic programming, remote sensing, polygon decomposition, coverage path planning (CPP), Boustrophedon paths, fixed wing UAV

## Abstract

In this paper, a new method for planning coverage paths for fixed-wing Unmanned Aerial Vehicle (UAV) aerial surveys is proposed. Instead of the more generic coverage path planning techniques presented in previous literature, this method specifically concentrates on decreasing flight time of fixed-wing aircraft surveys. This is achieved threefold: by the addition of wind to the survey flight time model, accounting for the fact fixed-wing aircraft are not constrained to flight within the polygon of the region of interest, and an intelligent method for decomposing the region into convex polygons conducive to quick flight times. It is shown that wind can make a huge difference to survey time, and that flying perpendicular can confer a flight time advantage. Small UAVs, which have very slow airspeeds, can very easily be flying in wind, which is 50% of their airspeed. This is why the technique is shown to be so effective, due to the fact that ignoring wind for small, slow, fixed-wing aircraft is a considerable oversight. Comparing this method to previous techniques using a Monte Carlo simulation on randomised polygons shows a significant reduction in flight time.

## 1. Introduction

The world’s population is exploding and, in order to keep up with the increasing levels of demand, we will have to produce 50% more food by 2050 [1]. In order to meet these demands, with the finite arable land we have available, it needs to be farmed far more efficiently. This can be achieved, in part, through the use of precision agriculture.

Technology has advanced significantly in precision agriculture over the years, giving farmers access to advanced farming tools, vehicles and data. Examples of the type of datas; weed distribution, soil pH, yield and crop health. This data, with the increasing availability of analytic technologies, mean that farmers can now use this data to provide actionable information, for example where and at what rate to apply water or fertilizer to a field. This data is normally gathered by hand by agronomists, dividing a field into sections and sampling. However, farms can be vast (Average US farm 175 ha [2]) so gathering any data by this meahod can be prohibitively time-consuming and expensive. Remote sensing has a possibility to change all of this. Remote sensing is where measurements are taken at a distance from the object.

Remote sensing on farms is primarily done using aerial images, using a number of spectral frequency ranges. In 1972, the Landsat-1 satellite was launched with an imaging package, capable of taking images in red, green, and two infra-red frequencies. This was to be the first of many satellites with the intention of performing remote sensing from space. Aerial imagery has a large number of uses—for example, soil moisture content [3], weed mapping [4] and yield calculation [5]. Unfortunately, the resolution of satellite images is low, using Landsat-1 as an example each pixel of an image was 79 m on the earth, while newer satellites (e.g., WorldView-2) boast a resolution of 0.5 m/pixel [6]. The lower resolutions of satellites are acceptable for some applications, however there are many that require sub centimetre Ground Sample Distances (GSD), which is impossible for a satellite. Satellite availability leaves much to be desired; depending on what orbit they are in, they can take a long time to be able to re-image a target, and images are easily blocked by haze or cloud.

One alternative is to collect images from manned aircraft that can fly underneath cloud cover. This is able to produce a finer GSD because photos are taken at lower altitude. However, these sorts of surveys are very expensive and are still constrained by weather, and their own operational safety concerns.

The final alternative is to take aerial photos and perform the remote sensing from a small unmanned aircraft equipped with an imaging system. Using an unmanned vehicle confers a number of advantages, discussed in the next section. Drone based remote sensing is becoming increasingly popular, and has been shown to be effective across a huge range of applications—for example, forestry and agriculture [7], coastal and environmental remote sensing [8]. In the area of precision agriculture, remote sensing has been shown to be a very successful tool in many areas like, disease mapping [9], or yield prediction in barley [10].

### 1.1. Advantages of UAV Surveying

With advances in sensor and embedded hardware technology, we are seeing a huge proliferation in the use of UAVs across many applications. This includes the essential sector of agriculture. It has been shown that performing remote sensing from a UAV is a cheap and effective way of gathering GIS, and spectral image data of arable land. This data can then in turn be turned into actionable information, e.g., variable rate fertilizer of pesticide application. This could in turn lead to the efficiency gains needed to keep feeding the ever increasing population of the Earth.

Low cost remote sensing can be performed by small fixed or rotary wing UAVs, taking multiple low altitude aerial images with a fixed or gimbaled imaging systems [11]. These have big advantages over using manned aircraft, as they are much cheaper, less restricted by weather and can be flown more regularly. To get a single high resolution image that covers an entire Region of Interest (ROI), a number of aerial images need to be stitched together to make what is known as an orthophoto. A Digital Elevation Model (DEM) of the ROI can also be produced. These post processing tasks can be performed using a large number of commercially available photogrammetry software packages, which use structure from motion algorithms to generate these outputs [12].

The task of generating a path that a vehicle’s coverage area will pass over all points of the ROI is called Coverage Path Planning (CPP). This can be mathematically formulated as a trajectory through the workspace *W* that will result in a set of *N* sensor readings: $Z_{1},Z_{2},\ldots ,Z_{k}$ such that the union of their area covers the whole of workspace *W*:

(1) $$\overset{k}{\underset{k=1}{⋃}}Z_{k}⊇W.$$

### 1.2. Literature Review

**Figure 1:**
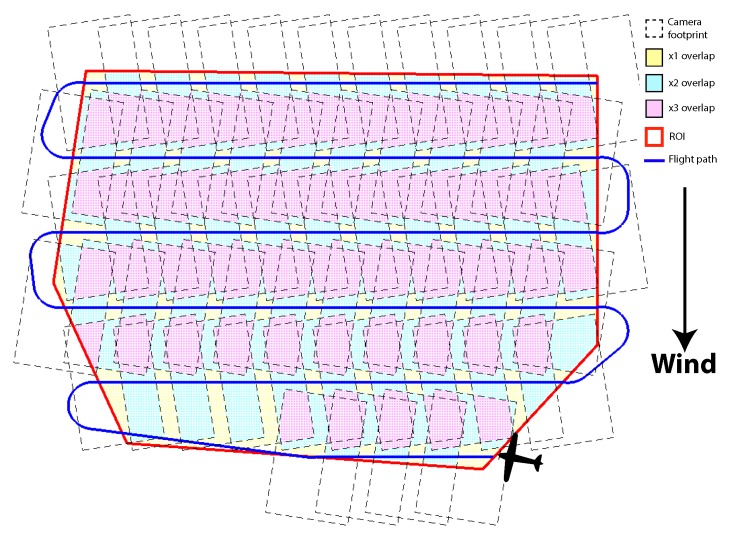
Example convex survey with a sidelap 40% and an overlap 40%, showing the Boustrophedon Coverage Path Planning (CPP), also showing the image footprints.

There are a large number of applications that use overage path planning, and so have been studied in detail in previous literature. A few example applications are lawn mowing, path planning for milling [13], indoor robotics [14], farm vehicle field coverage [15], fixed-wing surveys [16] and surveying using multi rotor aircraft [17]. The optimum path for a standard vehicle to cover a convex polygon is a simple back and forth sweep patten, presented in [18,19], and extended to 3D [20]. This is known as a Boustrophedon path. These paths also have the advantage of being very easy to generate with: knowledge of the ROI polygon, the heading of the sweeps, spacing between each sweep, which is calculated using the UAV’s sensor footprint, and required image overlap. An example Boustrophedon path in displayed in Figure 1, additionally showing each image footprint that needs to be taken to ensure complete coverage.

Most literature assumes that the path following for the vehicle is perfect, which for ground robots is a reasonable assumption. However, as fixed-wing aircraft are more susceptible to external disturbances, such as wind, and are limited in their manoeuvrability due to the fact that they need to maintain forward motion during flight, accurate path following is made much more difficult.

This sweep method is not used in [16]; they aim to generate a path in real time, which attempts to maximise the information gain image coverage. While this ensured total coverage, the flight time is increased and requires the camera to be gimbaled to compensate for the attitude of the aircraft. In addition to this, UAVs are not constrained to manoeuvring within the ROI like the ground vehicles described in most previous literature. They can, in fact, cut corners and overfly obstacles; therefore, this can be utilised in order to find faster paths.

A small study was conducted in Finland to attempt to quantify this. Some rough statistics on the general shape of Finish farm fields were generated in [15]. It reported that only 13% of fields are convex, and of those roughly 25% are simple shapes like circles, rectangles or triangles. Meaning that potentially 75% are complex shapes requiring smart path planning to find a fast path through the NP-hard solution space. Therefore, there is a real requirement for the development of efficient techniques for CPP of farm fields with complex shapes, as Boustrophedon paths are inadequate for these complex concave fields.

A number of papers solve this problem by using Boustrophedon Cell Decomposition (BCD) to separate the concave polygon into a number of convex polygons and then the Boustrophedon path is used to define paths for each individual cell. The problem of decomposing a polygon with holes into the minimum number of convex pieces is known to be NP-hard [21]. There are a number of decomposition techniques, such as triangular [22], visibility [23] and approximate decomposition [24]. A review of all these techniques are detailed in [18]. The BCD method is detailed in [25] where the exact decomposition is performed using trapezoidal decomposition [26]. Despite producing fewer cells than other decomposition methods [13,27], trapezoidal decomposition still tends to over-segment the ROI, resulting in wasted flight time transitioning between the cells, and, therefore, a recombination step is very important. BCD also doesn’t account for any survey or environmental factors and thus creates non-optimal cells. One method proposed is to use Dynamic Programming (DP) to recombine the cells [28]. Using DP ensures that the entire solution space is searched, meaning that the optimal solution for that search space is found.

In order to produce an optimal solution, an appropriate cost function needs to be defined, which is generally based around the aircraft’s flight time. Under ideal conditions, the path with the lowest Number of Turns (NT) will be the shortest [19,25].

The most common method to minimise turns is to simply align the sweep angles with the long axis of one of the decomposed cells, as this will increase the length of each leg of the path thus decreasing NT. An alternative method is to Minimise the Sum of the Altitudes (MSA) [28]. However, a fixed-wing aircraft’s ground speed, and time in a turn, depend heavily on its airspeed and the wind direction and speed. This could mean that NT is an inadequate optimisation parameter for fixed-wing UAV CPP under real-world conditions.

Small UAVs tend to be light and fly slow, and this makes them more vulnerable to the effects of wind. This is described in [19] in a simulated survey of an 1 × 0.6 km area, and wind extended their nominal 21 min flight by 6 min. This means that accounting for the wind in any kind of path optimisation is imperative; otherwise, the aircraft may not have the flight time to achieve a full survey. A flight time survey model for fixed wing aircraft in a steady uniform wind field was presented in [29]. It was also shown that wind will have a huge impact on flight time and taking it into account for planning a survey is essential.

### 1.3. Contributions

The aim of this paper is to generate survey flight paths for a survey UAVs in complex shaped ROIs, which takes into account environmental and operational factors not previously considered. In order to generate these paths, the complex ROI polygon needs to be decomposed into convex regions that can be covered by Boustrophedon paths. While the idea of this kind of decomposition is not completely novel, the significant additions and modifications in this proposed technique make it specifically tailored for fixed wing remote sensing, which have led to reduced survey times. One could extend this approach to other areas such as water-borne search and rescue in water currents.

One of the main contributions of this work also exploits the potential for the aircraft to fly outside the ROI in order to reduce the flight time. This is achieved by the inclusion of “optional” external cells which allows many decomposed cells to be recombined, which would otherwise create a concave polygon. As a result, a novel “bottom up” dynamic programming approach has been developed which improves computational efficiency dramatically compared to previous work. The work from [29] has been extended to prove that the minimum flight time exists when the survey direction is perpendicular to the wind.

A summary of the specific contributions from this paper is as follows:

- “Bottom up” DP approach speeds up optimal polygon decomposition.
- Optional cells external to ROI considered as part of decomposition to find alternate decompositions with lower flight times.
- Novel cost function for calculating flight time in the presence of wind.
- Mathematical proof of minimum flight time with the survey direction perpendicular to the wind.

**Figure 2:**
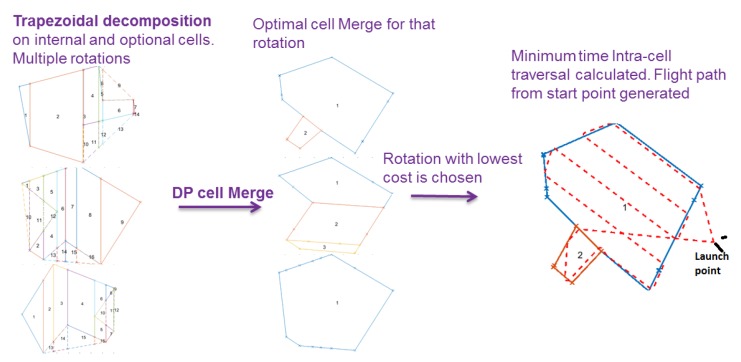
A summary of the major steps of the proposed technique, including trapezoidal decomposition, optimal cell merge, polygon rotation, then finally generating the full flight path.

A summary of the stages of the proposed technique is shown in Figure 2. First, the polygon will be decomposed using trapezoidal decomposition for multiple rotations of the polygon. This produces a large number of small cells that will be merged to make convex polygons, enabling the fastest coverage path plan in wind for each initial polygon rotation. The rotation with the lowest overall cost is chosen and the cell merge from this rotation will be used as the final polygon decomposition. Then, minimum time traversal path is planned around this decomposition to give the final path and waypoints to be used by the fixed-wing survey vehicle itself.

### 1.4. Report Structure

The rest of paper is organised as such; Section 2 presents the Boustrophedon path definition to guarantee coverage of a convex polygon ROI in wind. Section 3 describes how to decompose a concave polygon into a number of segmented convex polygons using trapezoidal polygon decomposition. This section also explains how the optional external cells are generated from the concave ROI’s convex hull. Section 4 details how many of the over-segmented cells can be recombined to give an optimal convex decomposition of the ROI for aerial surveying in wind. Section 5 explains how the actual flight path is generated by finding a minimum time traversal of all convex cells. In Section 6, a few example fields have a mission planned using the proposed method for illustrative purposes. Then, this method is compared to previous methods from literature on a number of real arable fields. Section 7 summarises the findings and concludes the work.

## 2. CPP in Convex Survey Regions

The CPP Boustrophedon paths consist of two different states of flight: the straight sweep paths when the photos are taken, and the turns used to transition to the next sweep.

**Figure 3:**
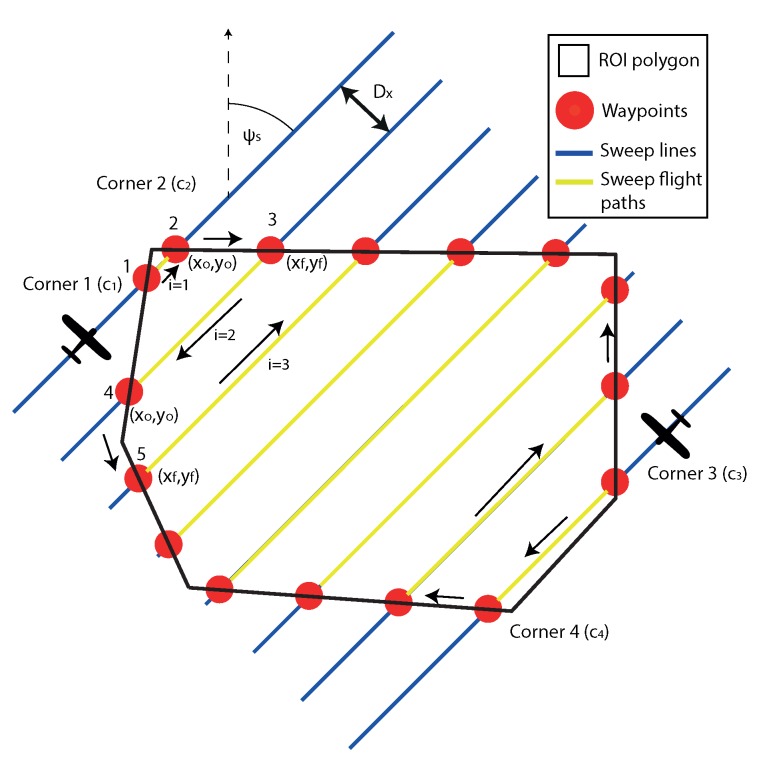
Sweep line intersections with the Region of Interest (ROI) polygon define the straight line waypoints. Showing the order of the traversal of all the sweeps in a convex survey. Additionally, it shows that the survey can start in one of four corners.

The start and the end of each sweep can be represented by waypoints, defined where the locations where the sweep lines intersect with the ROI polygon, as shown in Figure 3. Two waypoints represent a single survey sweep line with the coordinates $x_{w}^{i},y_{w}^{i}$, as there will be multiple sweeps, and *i* is the index:

(2) $$\begin{array}{cc} x_{w}^{i}=\left[\begin{array}{c} x_{o}^{i}\\ x_{f}^{i} \end{array}\right] & y_{w}^{i}=\left[\begin{array}{c} y_{o}^{i}\\ y_{f}^{i} \end{array}\right], \end{array}$$

where start indicates the start waypoint of the sweep, and end is the end waypoint of the sweep.

The waypoint, where the aircraft initially intercepts the sweep path is defined as [$x_{f},y_{f}$] with the heading in the direction of the sweep angle $\psi_{s}$, and the waypoint that defines the end of the sweep is [$x_{o},y_{o}$], at a heading of ($\psi_{s}$). There are four corners ($c_{k}=\left\{c_{1},c_{2},c_{3},c_{4}\right\}$) the aircraft can chose to start from; once selected, this will dictate the correct order of the waypoints to achieve the appropriate back and forth motions.

In Figure 3, a Boustrophedon path is generated at a sweep angle of 45${}^{\circ }$. This is clearly not optimal as some of the straight paths are extremely short and will include many more turns than required. A simple but highly effective way to select a sweep angle that minimises the number of turns is to align the sweep with the long axis of the polygon [25]. This is achieved by finding the minimum area bounding box that encloses the polygon. Then, the bounding boxes known long axis angle ($\psi_{bb}$) is used as the sweep angle.

The spacing between the sweeps is calculated to give a user defined sidelap between the images for a survey flight at a fixed altitude. The sidelap is set based on the requirements of the mission. For example, if high accuracy digital elevation models are needed, a high sidelap of >70% is required; however, if only the orthophotos is needed, the sidelap flown can be reduced to approximately 50%. The altitude to fly at is set by the required GSD, which is also based on the needed resolution of the application. This is calculated below:

(3) $$h=\frac{N_{x}GSD}{fov_{x}},$$

where *h* is the height of the aircraft above ground level, the units for GSD are $\frac{m}{pix}$, and $N_{x}$ is the number of horizontal sensor pixels. $fov_{x}$ is the horizontal angular field of view of the sensor. Then, the track spacing $D_{x}$ (also shown in Figure 3) can be calculated as:

(4) $$\begin{array}{c} D_{x}=2h\tan\left(\frac{fov_{x}}{2}\right)\left(1-w_{s}\right),\\ D_{y}=2h\tan\left(\frac{fov_{y}}{2}\right)\left(1-w_{o}\right), \end{array}$$

where $w_{o}$$w_{s}$ are the desired image overlap and sidelap between tracks, respectively. This makes the assumption that the images are taken orthogonal to the surface.

In addition to sidelap, there is also longitudinal overlap, and this is the same as sidelap, but it is the overlap of the images along the sweep path. The requirements for these are the same as sidelap. The distance between successive photos along each sweep path ($D_{y}$) is dictated by an equation analogous to $D_{x}$, also shown in Equation (4). Therefore, the frequency of photos can be calculated using the aircraft’s forward velocity. Most cameras and imaging systems have a cycle time between taking images due to limitations of processing time and memory write speeds. As an example, the Sony Nex 7 has a minimum time between photos of 0.7 s; a survey of a fixed wing flying at 15 m/s will lead to a minimum $D_{y}$ of 10.5 m. This parameter tends not to be a consideration as most cameras will take photos fast enough; however, if flying low or fast, this needs to be checked.

**Figure 4:**
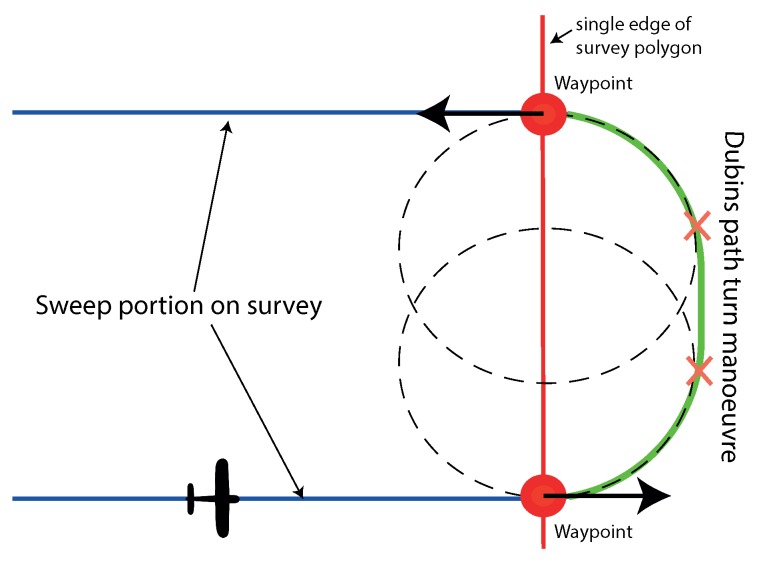
Turn manoeuvre between sweeps in zero wind using Dubins Paths. This demonstrates the fundamental setup for the survey model.

For the aircraft to transition from one sweep path to the next, it needs to perform a turn manoeuvre. These turns alternate between the left and right hand, and involves a turn of $180^{\circ }$ to its reciprocal heading. To complete the flight time model, this manoeuvre needs to be defined. This can be realised with the use of Dubins paths. A Dubins path is the shortest curve that connects two points in a 2D plane with a constraint on the curvature (in this case, aircraft maximum turn rate) [30]. A Dubins path is shown in Figure 4, and it completes the path between the adjacent tracks to give a continuous flight path. Figure 4 shows how these paths are constructed from two turn circles (whose radius is calculated from airspeed and maximum turn rate) positioned relative to the start and end points of the manoeuvre. All internal and external common tangents are found between the two circles, and only some of will form feasible flight paths. In the case of Figure 4, only a single external tangent exists that is feasible. All parts of the manoeuvre are simple geometric shapes, which lend themselves to easy length and flight time calculations.

### 2.1. Survey Flight Time Model in Wind

Wind can have a significant effect on small aircraft; the wind-speed experienced by a small UAV can easily be 50% of the airspeed. As a result, it is vital to account for wind for small survey UAVs. Wind will have a number of effects on a UAV survey. Firstly, the ground speed between opposing sweeps will be different. In addition to this, wind will have an effect on the alignment of the images; this is discussed in more detail later in this section. Finally, in the turns, Dubin’s curves assume zero wind; when performing the same manoeuvre in wind, the turn shape will be a geometric shape called a trochoid in the ground frame, while still being circular in the wind frame [30]. To achieve the same minimum distance path and hence minimum time path, the trochoidal turns are incorporated into Dubins framework; this then gives a path length and time that accounts for the wind in a turn manoeuvre at maximum turn rate.

This section details how to calculate the flight time of a fixed-wing aircraft surveying a convex polygonal area. This will be used in a later section to define the cost function used in the optimisation stage of this algorithm. The cost function developed will be henceforth referred to as Flight Time in Wind (FTIW). The wind field is assumed to be steady and uniform, as the survey is conducted over a relatively small area over a short time, making spacial and temporal variability minimal.

The wind will push the aircraft slowly off its course while flying along the straight sweep paths. To stop this, the aircraft must fly at a corrected heading, which will be slightly into the wind. This is in order to cancel out the velocity component of the wind to keep the ground velocity vector parallel to the sweep path. This heading correction is know as the Wind Correction Angle (WCA) ($\psi_{WCA}$). The WCA is derived from using the sine rule, with the wind triangle, shown below:

(5) $$\psi_{WCA}=\arcsin\left(\left(\frac{V_{w}}{V}\right)\sin\left(\psi_{wta}\right)\right),$$

where *V* and $V_{w}$ are airspeed and wind-speed, respectively, $\psi_{wta}$ is the wind to track angle, which is the aircraft’s heading relative to the wind, calculated by $\psi_{s}-\psi_{w}$, where $\psi_{w}$ is the wind direction, and $\psi_{s}$ is the sweep direction angle. Now, the WCA is known, the ground speed ($V_{g}$) and sweep flight time can be calculated. $V_{g}$ is the sum of the *x*-components velocity through the air mass and the *x*-component of the wind velocity, shown below:

(6) $$V_{g}=\left(V\cos\left(\psi_{WCA}\right)\right)+\left(V_{w}\cos\left(\psi_{wta}\right)\right).$$

Note the assumption that the small angles of WCA will mean that the rotation of the images coursed will not have any effect on the % sidelap. However, this can happen with WCA angles over 40${}^{\circ }$, which will only happen with perpendicular winds at $\frac{V_{w}}{V}$ of 0.65%, as calculated using Equation (5).

**Theorem** **1.** *A fixed wing UAV will have a lower flight time in the straight and level sweep portions of a Bousdophodon survey path, whose sweep angle is perpendicular to the wind, than a sweep angle directly into wind.*

**Figure 5:**
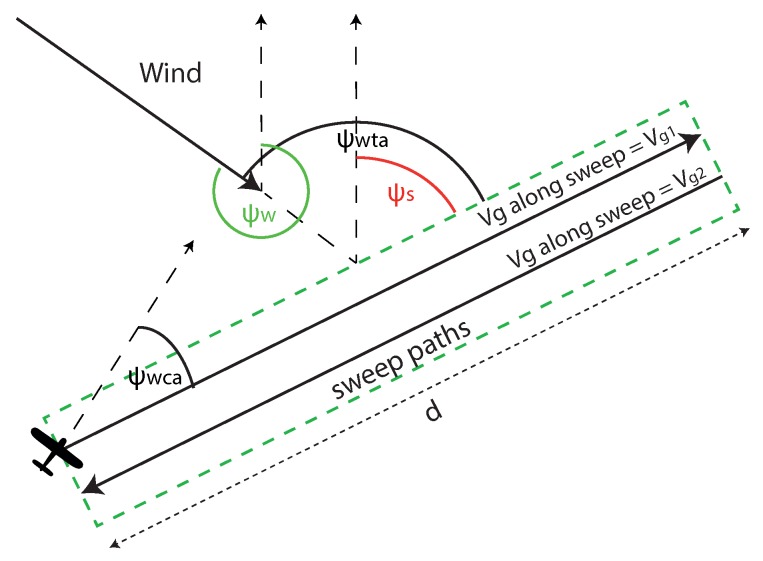
The illustration of a scenario designed to show that flying perpendicular to the wind is always faster than directly into it along the sweep portion of the survey.

**Proof** **of Theorem 1.** To prove that this is true, the most simple survey scenario will be assumed. This is a survey aircraft flying just two parallel tracks of the same length, one after the other, in the presence of a steady uniform wind field, shown in Figure 5. This represents a single back and forth survey path for coverage of a small rectangular ROI, the simplest example of a survey possible. If these two paths are rotated to a different relative angle to the wind $\psi_{wta}$, the distance travelled will not change. However, as the ground speed along each track changes in a nonlinear fashion with $\psi_{wta}$ the time optimal angle is not obvious. To prove the theorem, an equation for flight time along the two tracks need to be generated. By deriving the first and second derivative of the equation, we can show that $\psi_{wta}$ of 90${}^{\circ }$ will always be the maximum and 0${}^{\circ }$ will be the minimum. ☐

Equations to calculate the total time of these parallel tracks are generated using Equation (5) and Equation (6). The constant ground speed for each track is generated below:

(7) $$\begin{array}{c} V_{g1}=\left(V\cos\left(\arcsin\left(\beta \sin\left(\psi_{wta}\right)\right)\right)\right)+\left(V_{w}\cos\left(\psi_{wta}\right)\right),\\ V_{g2}=\left(V\cos\left(\arcsin\left(\beta \sin\left(\psi_{wta}+\pi \right)\right)\right)\right)+\left(V_{w}\cos\left(\psi_{wta}+\pi \right)\right), \end{array}$$

where $V_{g1}$ and $V_{g2}$ are the ground speed along the first and second track, which have ground track headings of $\psi_{wta}$, and the reciprocal $\psi_{wta}+\pi$, respectively. β is the wind to airspeed ratio $\frac{V_{w}}{V}$. $\psi_{wta}$ will in the interval (0,2pi]. This is because the aircraft will turn around after the first track and head back in the opposite direction.

The time taken to fly the straight and level tracks (not the turns) is shown below:

(8) $$t=d(\frac{1}{V_{g1}}+\frac{1}{V_{g2}}),$$

where *d* is the equal distance of both tracks, and *t* is the time taken.

By substituting Equation (7) in to Equation (8), a full equation for *t* will be given below:

(9) $$f\left(\psi_{wta}\right)=-\frac{2V{\left(\left(V_{w}^{2}\cos{\left(\psi_{wta}\right)}^{2}+V^{2}-V_{w}^{2}\right)/V^{2}\right)}^{0.5}}{\left(V^{2}-V_{w}^{2}\right)}.$$

The $\psi_{wta}$ angles which minimise survey time can be found by ascertaining where the first derivative of Equation (9) is zero ($f^{\prime }\left(\psi_{wta}\right)=0$). Its derivative can be seen below:

(10) $$f^{\prime }\left(\psi_{wta}\right)=\frac{-(2V_{w}^{2}\cos\left(\psi_{wta}\right)\sin\left(\psi_{wta}\right))}{\left(V\left(V^{2}-V_{w}^{2}\right){\left(\left(V_{w}^{2}\cos{\left(\psi_{wta}\right)}^{2}+V^{2}-V_{w}^{2}\right)/V^{2}\right)}^{0.5}\right)}.$$

By equating this equation to zero and simplifying $2V_{w}^{2}cos\left(\psi_{wta}\right)sin\left(\psi_{wta}\right)=0$, all solutions can be calculated using:

(11) $$\psi_{wta}=\frac{n\pi }{2}.$$

This gives critical points at $0,\pi /2,pi,\frac{3\pi }{2}$ in the given interval which are for the following values of *n*n=[1,2,3,4]. These are both the maximum and minimum points. By taking the second derivative of Equation (9) and substituting the critical $\psi_{wta}$ angles given above into $f^{\prime \prime }\left(\psi_{wta}\right)$, we can find if each point is a minimum or a maximum. The result will be −ve for maximum and +ve for minimum. $f^{\prime \prime }\left(\psi_{wta}\right)$ is not included here due to its complexity.

**Figure 6:**
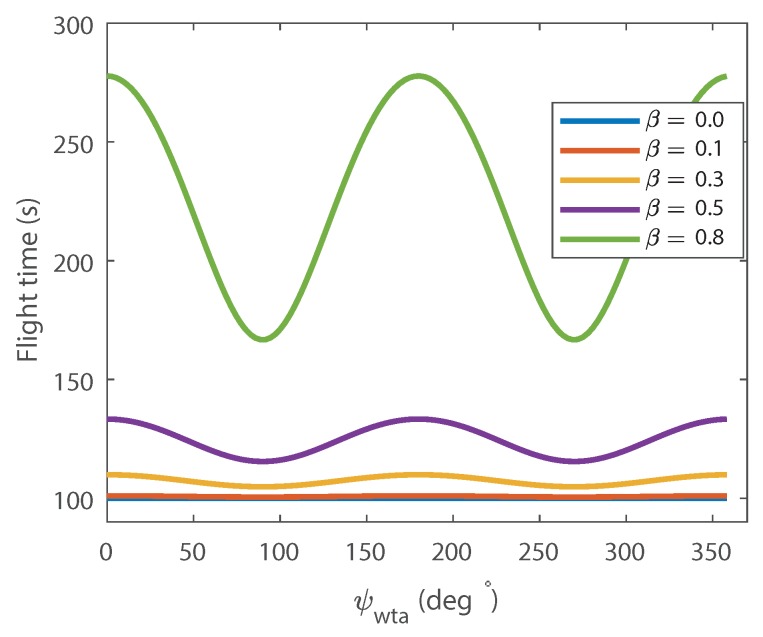
Total flight time for the portion of the flight along two parallel 500 m tracks as defined in Figure 5 for a range of wind/airspeed ratio.

The results of these substitutions: $f^{\prime \prime }\left(0\right)=-ve$, $f^{\prime \prime }\left(\frac{\pi }{2}\right)=+ve$, $f^{\prime \prime }\left(\pi \right)=-ve$, $f^{\prime \prime }\left(\frac{3\pi }{2}\right)=+ve$. This means that the minimum points are $\frac{\pi }{2}$ and $\frac{3*\pi }{2}$, and the maximum to be 0, π. This proves that flying with perpendicular to the wind i.e., $\psi_{wta}$ angles of $\frac{\pi }{2}$ and $\frac{3\pi }{2}$ minimise the survey time along the sweep tracks, whereas flying parallel to the wind increases flight time, by how much depends on β. This can clearly be seen in Figure 6 which contains plots generated from Equation (9) that show the total flight time to fly both 500 m parallel tracks in a range of wind-speeds.

### 2.2. Trochoidal Turn Paths

The time taken in the turns between sweep paths needs to be calculated. To achieve this, the path taken needs to be defined. However, wind needs to be taken into account. Dubin’s style path planning is used to define these turns; however, in wind, the previous circular turns will be not be circular due to the wind. When a circle is drawn in a steady moving frame of reference, it becomes a shape called a trochoid. It was shown in previous literature that this Dubins style path planning can be applied in wind by finding the feasible tangents between the two trochoids at the start and the end of the turn manoeuvre [29,31].

There are a number of feasible paths that can be generated, and the two turns in the manoeuvre are either right *r* or left *l*, joined with a straight *s* tangent between them with four combinations lsl lsr rsr rsl. Due to the nature of the Boustrophedon path, all turns will be the same side turns, leaving two possibilities rsr, lsl. If it turned in different directions, it would imply that the aircraft would need to turn more than 180${}^{\circ }$. It makes the problem simpler, as there is an analytical solution to the angle of tangency for the same side turns, as opposed to different side turns, which requires a root finding technique to solve.

**Figure 7:**
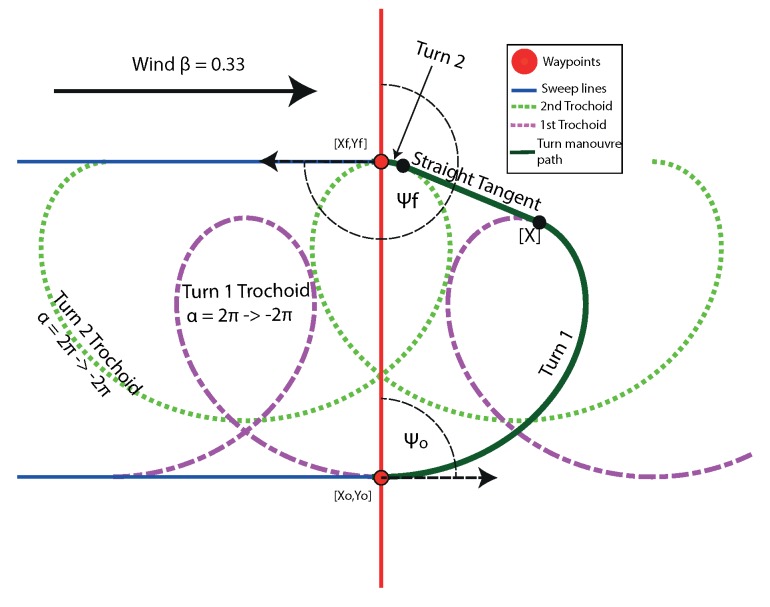
From Figure 4, showing the path layout for the survey model in wind using trochoidal turn paths in addition to the the full trochoids for α of 2π→−2π, where portions of these are used to generate the paths. In this example, the aircraft is flying sweeps parallel to the wind direction, known from Theorem 1 to be the least efficient.

Figure 7 shows the same turn from the Boustrophedon path as Figure 4; however, the circular turns have been replaced with trochoids to give a similarly smooth trajectory while in wind. Equation (12) shows the parametric equation for a trochoid. It has been rotated in order to put the equation into the trochoidal frame with subscript, *t*, and this means that everything is rotated to make the *y*-axis in line with the direction of wind, which is why the wind term β only appears in the *y*-axis in Equation (12):

(12) $$\begin{array}{c} x_{t}=-Rcos\left(\alpha \right),\\ y_{t}=Rsin\left(\alpha \right)+R\beta \alpha , \end{array}$$

where *R* is the turn radius, calculated from $\left(R=\frac{V}{\dot{\psi }}\right)$, and α is the angle subtended in the turn.

The construction of these trochoidal turn paths can be performed by knowing the start [$x_{o},y_{o}$] and end point [$x_{f},y_{f}$], the initial ($\psi_{o}$) and final headings ($\psi_{f}$) of the manoeuvre, the airspeed (*V*), turn rate ($\dot{\psi }$) and wind-speeds.

The calculations to achieve this are laid out in [29,31].

### 2.3. Full Survey Model

In order to find the total flight time over the full convex survey, the calculations for time in the turns and on the straight level sweep paths need to be combined in to $t_{conv}^{k}$, which is the time for a complete convex polygon at the *k* starting corner. Firstly, the time along each sweep path needs to be summed. As all the tracks are parallel, the aircraft’s heading will be either $\psi_{s}$ or its reciprocal when it is flying in the opposite direction. All the times for each sweep is summed to give the total time in the sweeps $t_{s}$ below, where $n_{s}$ is the total number of sweep paths:

(13) $$t_{s}=\sum_{i=1}^{n_{s}}\left\{\begin{array}{cc} \mathrm{if}\;i\;\mathrm{odd} & \left(\frac{∥x_{w}^{i}-y_{w}^{i}∥}{V\cos\left(\arcsin\left(\beta \sin\left(\psi_{wta}\right)\right)\right))+(V_{w}\cos\left(\psi_{wta}\right)}\right),\\ \mathrm{if}\;i\;\mathrm{even} & \left(\frac{∥x_{w}^{i}-y_{w}^{i}∥}{V\cos\left(\arcsin\left(\beta \sin\left(\psi_{wta}+\pi \right)\right)\right))+(V_{w}\cos\left(\psi_{s}+\pi \right)}\right). \end{array}\right.$$

The time for the full turn manoeuvre is found by summing the time in each turn:

(14) $$t_{t}=\sum_{i=1}^{n_{s}-1}t_{1}+t_{2}+\left(\frac{\sqrt{{\left(x_{w2}^{i}-x_{w1}^{i+1}\right)}^{2}+{\left(y_{w2}^{i}-y_{w1}^{i+1}\right)}^{2}}}{V\cos\left(\arcsin\left(\beta \sin\left(\arctan\frac{x_{w}^{i+1}-x_{w}^{i}}{y_{w}^{i+1}-y_{w}^{i}}\right)\right)\right)+V_{w}\cos(\arctan\frac{x_{w}^{i+1}-x_{w}^{i}}{y_{w}^{i+1}-y_{w}^{i}})}\right),$$

where $t_{1}$ and $t_{2}$ are the times in the turns and the third term is the time in the straight portion of the turn manoeuvre. These are generated using equations laid out in [29,31]. This can be achieved using the inputs [$x_{0},y_{0}$] [$x_{f},y_{f}$], and angles $\psi_{o}$ and $\psi_{f}$. [$x_{0},y_{0}$] [$x_{f},y_{f}$] are equivalent to [$x_{w2}^{i},y_{w2}^{i}$] [$x_{w1}^{i+1},y_{w1}^{i+1}$], respectively. The trochoid calculations are performed in the wind frame, thus the points need to be rotated by $-\psi_{w}$, shown in Equation (15). The angles $\psi_{o}$ and $\psi_{f}$ are aligned with $\psi_{s}$ or the reciprocal angle, and this relationship depends on the sweep index being odd or even, shown in Equations (16) and (17):

(15) $$\left[\begin{array}{c} x_{t0}\\ y_{t0} \end{array}\right]=\left[\begin{array}{cc} \cos(-\psi_{w}) & -\sin(-\psi_{w})\\ \sin(-\psi_{w}) & \cos(-\psi_{w}) \end{array}\right]\left[\begin{array}{c} x_{0}\\ y_{0} \end{array}\right],$$

(16) $$\psi_{0}=\left\{\begin{array}{cc} \mathrm{if}\;i\;\mathrm{is}\;\mathrm{odd} & \psi_{s},\\ \mathrm{if}\;i\;\mathrm{is}\;\mathrm{even} & \psi_{s}+\pi , \end{array}\right.$$

(17) $$\psi_{f}=\left\{\begin{array}{cc} \mathrm{if}\;i\;\mathrm{is}\;\mathrm{odd} & \psi_{s}+\pi ,\\ \mathrm{if}\;i\;\mathrm{is}\;\mathrm{even} & \psi_{s}. \end{array}\right.$$

The final full flight time taken to fully cover a convex polygon from the starting corner, *k*, is simply the addition of the time summations in the turns and along the sweeps:

(18) $$t_{conv}^{k}=t_{s}+t_{t}.$$

## 3. Cell Decomposition

In cases where the ROI is concave, the polygon will need to be initially decomposed into a number of convex polygons. There are a number of uses for these decomposition techniques, for example: 3D model mesh generation, robotic motion planning and computational geometry. Robotic motion planning is achieved by decomposing an area into a set of geometric shapes, whose centre can be used as potential waypoints for finding the fastest route [32]. The general aim in the development of these algorithms is to make them run as fast and as efficiently as possible to minimise computational time.

### 3.1. Trapezoidal Decomposition

**Figure 8:**
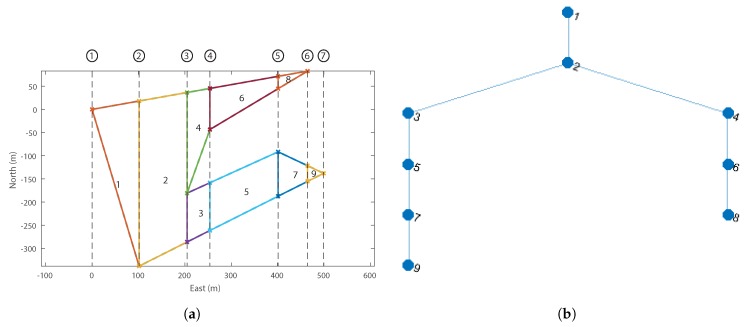
Example trapezoidal decomposition. (**a**) trapezoidal decomposition of polygon, showing vertical slice lines generated at each vertex; (**b**) cell connectivity graph of the example trapezoidal decomposition, where each node represents a decomposed cell, and each edge a cell adjacency.

Trapezoidal decomposition was chosen as the initial decomposition technique. This technique was chosen as it offers a number of advantages over other methods. By far, the most important advantage is the shape; a trapezoid is a much more efficient geometric shape to cover in the Boustrophedon paths described in Section 2. This is because the long thin trapezoids that are generated by the algorithm require significantly fewer turns of the survey aircraft to provide complete photo coverage than a triangle from example. The number of cells also needs to be kept to a minimum to speed up the recombination optimisation because this will lower the number of options. Trapezoidal decomposition tends to generate fewer cells than triangulation, or an approximate decomposition techniques. A good description of the general workings of trapezoidal decomposition is detailed in [26]. Shown in Figure 8a is a polygon decomposed using trapezoidal decomposition. The vertical sweep lines whose intersections with the polygon define the vertices of all the decomposed cells.

Using the specific trapezoidal decomposition technique laid out in [33] a solution can be found in $O(n_{v}\log\left(n_{v}\right))$ time, where $n_{v}$ is the number of vertices. This means high vertex count polygons will have acceptable computational time. Another important output of the initial decomposition is the cell connectivity graph, which will be used in the recombination phase. Knowledge of the cell adjacency is required to define a search space, but more importantly to identify which cells can be combined. Using the same polygon example, the adjacency graph is shown in Figure 8b.

**Figure 9:**
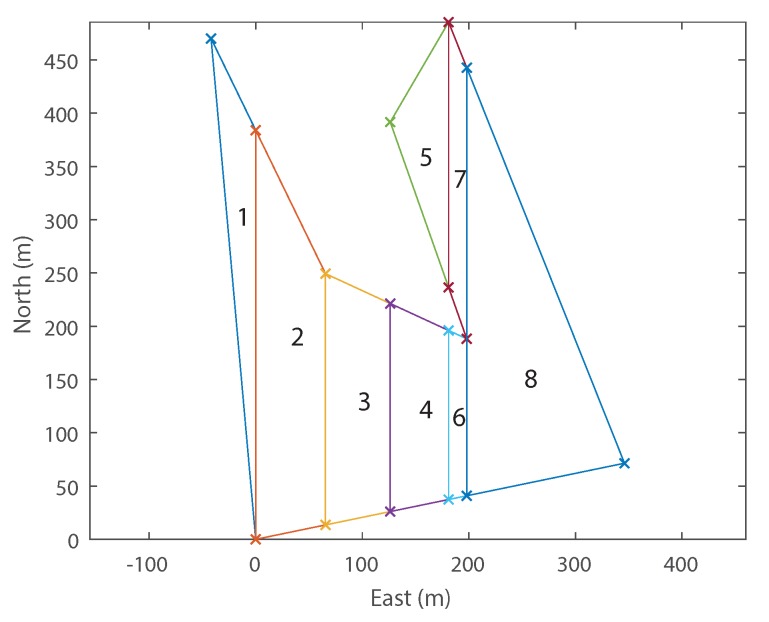
Example initial trapezoidal decomposition run on the example polygon from Figure 8a rotated by 90${}^{\circ }$.

As the slices are always vertical, the final decomposition for a particular polygon depends on its initial rotation. If the polygon is rotated, decomposed, and then rotated back to its original orientation, then an infinite number of decomposition possibilities exist. This means that there could be different and possibly more preferable decompositions at other rotation angles. An example trapezoidal decomposition is performed on a rotated version of the example polygon in Figure 9. Without consideration of wind, the optimal path is found by simply using the longest axis of the cell and minimising the number of turns. However, it has also been shown (Section 2.1) that flying perpendicular to wind is also advantageous. Therefore, the inclusion of wind results in two competing factors, meaning that a larger search space is necessary to find a preferable solution.

Each vertex of the polygon must be rotated about polygon rotation angle $\psi_{p}$. Let *P* be a polygon with $n_{v}$ verities $\nu =\{v_{1},v_{2},\ldots ,v_{n_{v}}\}$, then *P* can be rotated as show in Equation (19), where $P_{r}$ is the rotated Polygon, *P*, and $\psi_{p}$ is the angle of rotation.

(19) $$P_{r}=\left[\begin{array}{cc} \cos\psi_{p} & -\sin\psi_{p}\\ \sin\psi_{p} & \cos\psi_{p} \end{array}\right]P.$$

### 3.2. External Cell Decomposition

Unlike ground-based robots, the path of UAVs is not generally constrained to be completely within the ROI (unless there are any no-fly zones). They can fly over awkward corners and transfer between one convex cell survey to others by a direct route. This ability has been completely over looked in previous literature, so we propose a method to extend trapezoidal decomposition to include potential external cells that might shorten overall flight time.

These external cells need to be found and generated. The method to achieve this is based around the concave polygon’s convex hull. Given a set of points, its convex hull is the subset of points in Euclidean space that has the smallest set of points that create a convex polygon. In effect, a convex hull is the most simple convex polygon that contains all vertices of the concave polygon. The technique used from [34] is summarised below:

- determine the four external points of the set, discarding all points interior to the convex quadrilateral they form,
- break points into four regions that can be solved separately,
- use vector crossproducts to determine feasible convex paths in each sub region.

**Figure 10:**
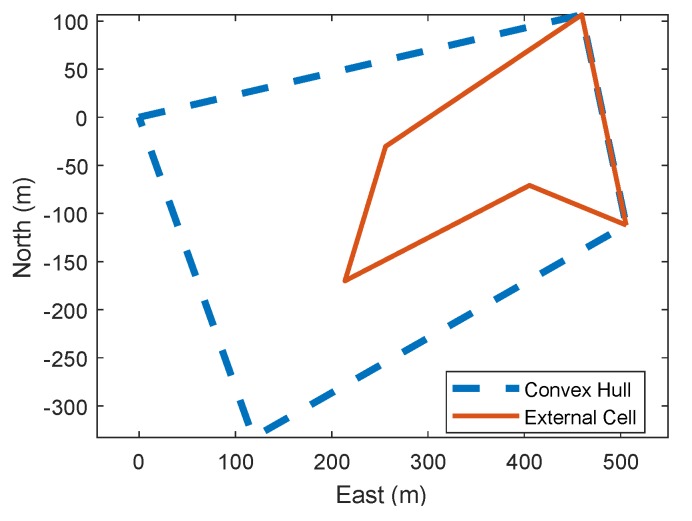
Convex hull, and optional external cells from the example concave polygon.

**Figure 11:**
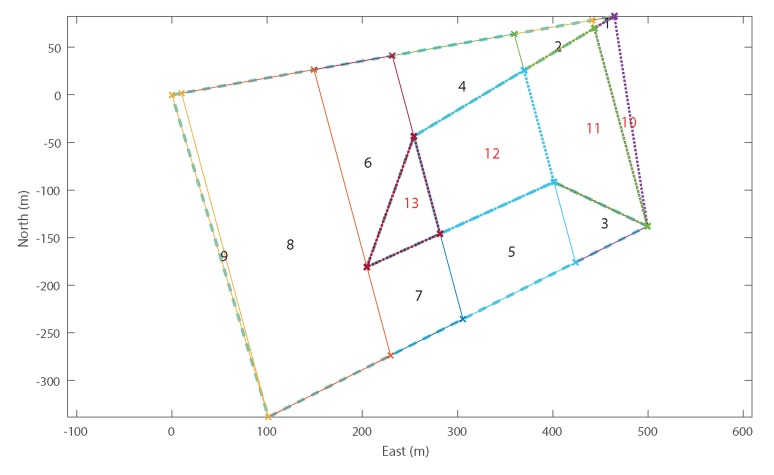
Full trapezoidal decomposition of ROI and optional external polygon.

**Figure 12:**
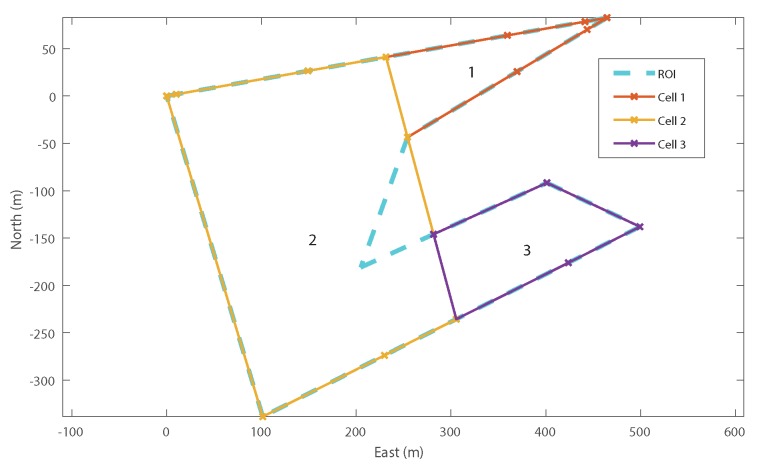
Cell merge preformed on the decomposed cells generated for Figure 11, where the merged cell number 2 contains the shortcut granted by combining normal and optional cells to make for a more efficient survey.

Shown in Figure 10 is the convex hull of the previous example ROI at $\psi_{p}$ of 345${}^{\circ }$. The convex hull of a polygon represented by $P_{chull}$. By performing a polygon subtraction $P-P_{chull}$, the external optional polygon is generated, and this is also shown in the figure as the solid red polygon. A polygon subtraction is when the area of one polygon is removed from another polygon. The algorithm to preform this task is laid out in [35]. The external polygon can then, itself, be decomposed to give all the optional cells using trapezoidal decomposition, shown in Figure 11. As the ROI and the external polygon share vertices, the decomposed cells will always line up with each other in the direction of the vertical slice from the trapezoidal decomposition. This means that the cell merge algorithm will generate more long, thin convex polygons, which are more conducive to quick surveys. Shown in Figure 12 is an example recombination between cell 9, 8, 6, 7 and optional cell 13. This gives a large single convex area much better for surveying then the three cells that would be left if there were no optional cells.

## 4. Cell Recombination Using Dynamic Programming

Dynamic Programming (DP) is a technique that can be used to solve complex problems by breaking them down into simpler sub-problems. The full solution can then be built by optimally combining the solutions to the smaller problems. Like “brute-force” methods, DP examines every single possible solution and therefore guarantees the optimality of the solution. However, DP offers the advantage that solutions to the simpler problems can be stored or “memo-ized”, meaning that the solutions to identical sub-problems can be re-used, resulting in considerable computational time savings if there is significant overlap of sub-problems. The cell recombination problem is highly suited to DP optimisation, due to the fact that it exhibits “optimal substructure”. This means that it can be solved optimally by breaking it down into smaller sub-problems and then using a recursive algorithm to compare the optimal solution to each of the sub-problems with the solution to the area as a whole (see Equation (20))

(20) $$J_{\pi }\left(G\right)=\min\left\{C\left(G\right),\min_{k}\left[J_{\pi }\left(G_{1}^{k}\right)+J_{\pi }\left(G_{2}^{k}\right)\right]\right\}.$$

$J_{\pi }\left(G\right)$ represents the optimal cost of searching region, *G*, as the minimum of either the simple cost, C(G), of searching the entire region or the sum of the optimal costs of searching any two sub-regions, $G_{1}^{k}$ and $G_{2}^{k}$, which combine to cover the entire search area. This algorithm can then be applied recursively by solving the two sub-problems, $J_{\pi }\left(G_{1}^{k}\right)$ and $J_{\pi }\left(G_{2}^{k}\right)$, using the same equation. The cost, C(G), of searching region *G* is defined as the flight time Equation (18) if the polygon is convex, otherwise it is given a very high penalty cost (e.g., infinite) (see Equation (21))

(21) $$C\left(G\right)=\left\{\begin{array}{cc} t_{conv}^{k}, & \mathrm{if}\;\mathrm{polygon}\;\mathrm{is}\;\mathrm{convex},\\ \infty , & \mathrm{otherwise}. \end{array}\right.$$

Dynamic programming calculates the optimal recombination by searching every possible partition of the set and as a result is still very computationally intensive. Huang et al. [28] have applied this algorithm directly in a “top-down” approach; this results in an exponential algorithm which requires slightly less than $O\left(c^{n}\right),\left(c>1\right)$ time, where *n* represents the number of cells in the decomposed search area.

However, in this work, the dynamic programming formulation used is a “bottom-up” approach beginning with a single starting cell and building the complete search area one cell at a time. This approach has been found to be more efficient due to the ability to immediately discount certain recombination patterns, such as those which would form a concave cell. In this case, the sub-region is assigned an infinite cost and all subsequent recursions from this branch are not calculated. From extensive testing of real and randomly generated fields, the optimisation time has been reduced to approximately $O\left(c^{n/2}\right),\left(c>1\right)$.

### 4.1. Bottom-Up DP Formulation

**Figure 13:**
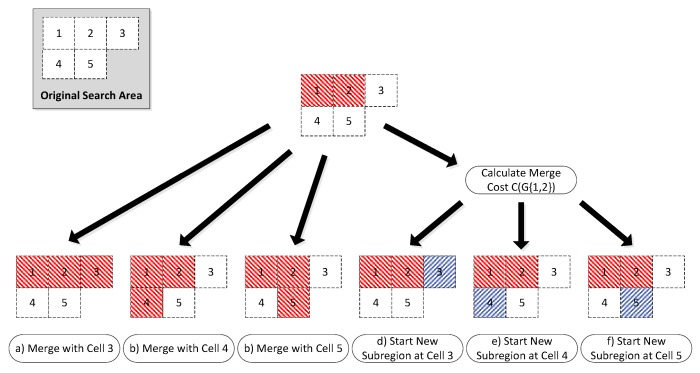
“Bottom Up” Dynamic Programming Solution Formulation—showing a single cell merge step, where each adjacent cell is either merged or a new subregion is started.

Mathematically, the bottom-up formulation is the same Equation (20); however, algorithmically, the order of calculations is performed slightly differently, resulting in a lower memory usage and significantly fewer unneeded computations of the cost function. Instead of starting with the simple cost, C(G), of searching the entire region and then gradually breaking the area up into smaller sub-regions, $G_{1}^{k}$ and $G_{2}^{k}$, the bottom-up approach begins with a single cell and gradually builds the search graph by either recombining neighbouring cells or remaining unmerged (see Figure 13).

From any initial point, a number of potential actions can be performed. Either the cell may merge with any *adjacent* cell (Figure 13a, b or c), or the current merge may be declared complete and a new merge sequence may begin with any other *adjacent* cell in the search area (Figure 13d, e or f). Each of these actions will result in a new starting point and the algorithm works recursively until every *internal* cell in the search area is accounted for.

At each step, the cost of the current merge is calculated and memo-ized for potential re-use in future steps. For example, the subsequent steps from Figure 13e,f will both result in recombining G{4,5} resulting in C(G{4,5}) being required. The first time this calculation is encountered, it will be calculated and stored, and any subsequent times it will simply be recalled from memory.

The bottom-up approach allows for external cells to be *optionally* included much more efficiently. With a top-down approach, it would be necessary to initialise the problem multiple times for every combination of potential external cells, some of which may result in no additional viable solutions. With a bottom-up approach, the algorithm is allowed to continue until it finds every possible solution that accounts for every *required* (internal) cell, whether or not external cells are included in the solution. The recursive function described is laid out in pseudocode in Algorithm 1.

**Algorithm 1** My algorithm
  1:
**procedure** DP Recombination
  2:  **initialise** (  3:  CurrentSuperCell=1  4:  CurrentMerge:=1)  5:  **function** recursiveFunction(data, CurrentMerge, CurrentSuperCell)  6:  **end function**  7:  %Step 1. Merge with Any Neighbour  8:  List All Neighbours to CurrentSuperCell  9:  **for** ForEachNeighbour **do**10:   NewMerge:=CurrentMerge+Neighbour11:   **if** NewMerge doesn’t already exist in data **then**12:    data.createNode(NewMerge)13:    data.buildEdge(NewMerge←CurrentMerge,0)14:    NewSuperCell=CurrentSuperCell+Neighbour15:    recursiveFunction(data, NewMerge, NewSuperCell)16:   **end if**17:  **end for**18:  %Step 2. Calculate Cost of Current Merge (inc. memo-ization)19:  **if** Costs of CurrentSuperCell already been calculated **then**20:   NewCost=data.Cost(CurrentSuperCell)21:  **else**22:   NewCost=fCost(CurrentSuperCell)23:   data.Cost(CurrentSuperCell)=NewCost24:  **end if**25:  %Step 3. Start New Merge with Any Required Cell26:  List all new internal cells not already included in CurrentMerge27:  **for** Each NewInternalCell **do**28:   NewMerge:=CurrentMerge+NewInternalCell29:   **if** NewMerge doesn’t already exist in data **then**30:    data.createNode(NewMerge)31:    data.buildEdge(NewMerge←CurrentMerge,NewCost)32:    NewSuperCell=NewInternalCell33:    recursiveFunction(data, NewMerge, NewSuperCell)34:   **end if**35:  **end for**36:  %Step 4. Create Edge to Exit37:  **if** all internal cells are already included in CurrentMerge **then**38:   buildEdge(Exit←CurrentMerge,NewCost)39:  **end if**40:  **return** data41:
**end procedure**



### 4.2. Example Recombination

**Figure 14:**
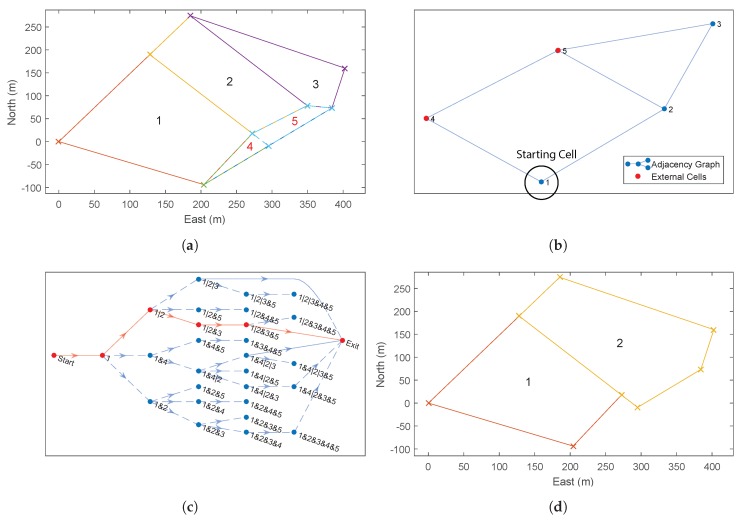
“Bottom Up” Dynamic Programming Recombination Example. (**a**) decomposition of ROI with two external polygons; (**b**) adjacency graph of decomposed polygons; (**c**) full search network using dynamic programming (Notation: “1|2” denotes that cells one and two are separate polygons, “1&2” denotes that they have been recombined); (**d**) recombined ROI into two polygons. Note that only one (#5) of the two optional cells has been chosen by the optimiser.

Figure 14 shows an example recombination of a decomposed ROI with three internal cells and two external cells (Figure 14a). In addition to the co-ordinates of the decomposed cells, the optimiser is also provided with an adjacency graph which shows how the cells can be merged (Figure 14b) and the list of external cells (cells 4 and 5, highlighted in red).

The first step of the optimiser is to build up the search network shown in Figure 14c. For decomposed ROIs with a large number of cells, this search graph can become very large, so, for legibility, this example has been deliberately kept very small using just five cells. Using a recursive algorithm, the optimiser builds this graph starting with the lowest (displayed) branch from the start node on the left-hand, side moving towards the exit node on the right-hand side. Once the optimiser reaches a node from which no more forward branches are possible (such as the exit node), the optimiser takes one step back and tests for alternative solutions. This recursion continues until all possible branches have been exhausted. Any node which includes all internal cells is allowed to branch directly to the exit node and therefore represents a possible final solution.

Detailed examination of Figure 14c highlights the effect of some of the optimisations applied to the recombination process. Firstly, it can be seen that, in the first stage, the optimiser examines recombination with both adjacent cells, 2 and 4 (Rule 2), but only examines starting a new polygon with cell 2. This is because cell 4 is an *optional* external cell (Rule 3). The net result of this rule is that “1|4|...” is never examined; however, cell 4 may still be included subsequently if it merges with a required cell later on, for example “1|2&4&5”.

Secondly, it can be seen that node “1|2|3”, at the top of the diagram has a branch going straight to the exit. This is because this solution already contains all of the *required* internal cells and therefore represents a solution in and of itself (Rule 5).

In addition, although node “1&2&3” also contains all of the required cells, this node does not have a branch to the exit node. In fact, this node only has branches which perform further merging with other cells. This is because the recombination of G{1,2,3} would result in a concave cell, for which the cost has been defined as infinite. Therefore, the optimiser does not attempt to calculate the cost of these further branches (Rule 4).

Finally, there are no branches from “1&2&3&5”. As for “1&2&3”, this sub-region is concave and therefore must be merged with additional cells to create a viable sub-region. In this case, the only viable additional cell is cell 4; however, the resulting sub-region “1&2&3&4&5” has already been found (branched from “1&2&3&4”) and therefore it is unnecessary to calculate it again (Rule 1). Similarly, there are no branches from “1&2&4&5” and “1&2&5”.

The efficiency of the DP algorithm can be compared to brute force methods by looking at the number of solutions calculated, compared to the total number of partitions of a set [36]. The net effect of these optimisations is that the number of nodes at each stage is minimised as soon as possible, resulting in significantly fewer calculations. As a result, there are only five (out of a maximum 87) possible partitions of the set examined. For comparison, the top-down approach inherently examines only neighbouring cells (Rule 2) but has no other optimisations. For this problem, it would have identified 39/87 partitions (see Table 1). The optimised “bottom-up” formulation removes 32 of these partitions due to concavity and an additional two due to external cells not required.

Figure 14d shows the optimised recombination of the decomposed cells. Note that *external* cell 5 has been included in order to allow cells 2 and 3 to be merged and generate a convex sub-region, but cell 4 has been rejected, resulting in two final sub-regions, G{1} and G{2,3,5}.

### 4.3. Polygon Convexity

The convexity of a polygon needs to be determined in order to test if each new cell merge during the DP optimisation from the above section is suitable for a convex survey.

Convex polygons have a number of unique properties distinguishing them from concave polygons. In a convex polygon, the sum of all interior angles of a polygon will be less than 180${}^{\circ }$. In addition, if each edge along the polygon is evaluated in sequence, the direction of rotation of the next edge will always be in the same direction if the polygon is convex. This final property was used in [37] to create an effective algorithm for finding polygon convexity called the gift wrapping algorithm.

**Figure 15:**
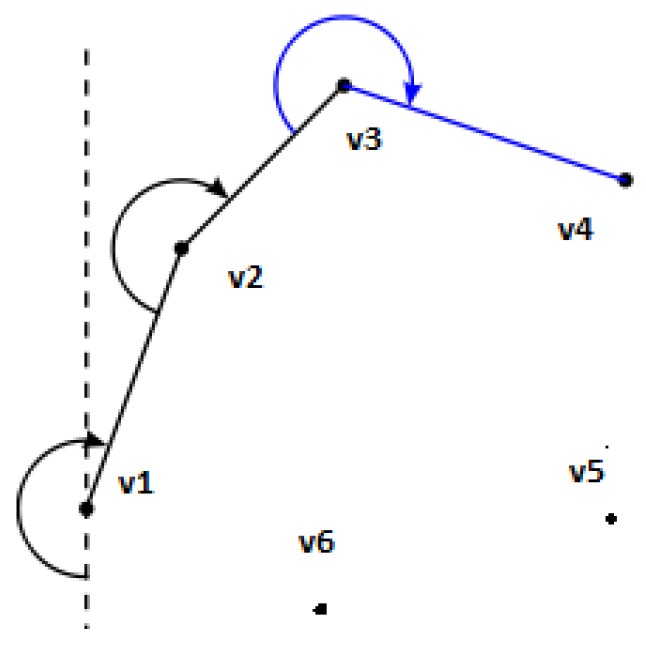
The gift wrap algorithm successively assessing each vertex around a polygon for convexity.

If all the vertices are evaluated in a clockwise direction, they can be individually assessed for convexity. This can be seen in Figure 15. If all vertices are convex, then the whole polygon is convex. The direction of rotation of each is assessed by taking the cross product as shown below. If the result is +ve the vertex is convex, −ve indicates a concave vertex, and 0 means that the vertices $v_{i-1},v_{i},v_{i+1}$ form a straight line:

(22) $$v_{i-1}v_{i}\times v_{i}v_{i+1}=\left\{\begin{array}{cc} >0, & \mathrm{convex}\;\mathrm{vertex},\\ =0, & \mathrm{collinear}\;\mathrm{vertices}\;(v_{i}\;\mathrm{convex}),\\ <0, & \mathrm{concave}\;\mathrm{vertex}. \end{array}\right.$$

## 5. Multi-Cell Routing and Flight Time Calculation

Thus far, all flight time calculations have been performed individually on each convex polygon, enabling the problem to be split into independent sub problems. While this gives us a good approximation for how long the flight will be, the transition between each merged cells has not been taken into account. The full survey path to be flown, including transitions, now needs to be defined. In addition, the full survey flight time in wind for a given decomposition is needed. This is needed in order to give a realistic assessment between the different cost functions available to the DP algorithm. This full flight path will include a launch point (start and end point of the survey), the individual decomposed convex polygon survey paths, and feasible transitions paths between them. Currently, the outputs from the FTIW, NT and MSA cost functions are seconds, number of turns, and meters, respectively, therefore, a direct comparison between them is impossible.

The fastest route can be found from the minimum weight path of a weighted directed graph, where the weight of each edge is the time taken to perform the manoeuvre the edge represents. This will be either be the time to fly the transfer path between cells, or the time taken to perform a convex survey.

The initial join point for a convex survey can be in one of the four corners (*C*) of convex polygon (*P*), where *j* is the index of the cell and the order of the corner numbering is clockwise:

(23) $$C^{j}\left(P\right)=\left[\begin{array}{cc} c_{1}^{j} & c_{2}^{j}\\ c_{3}^{j} & c_{4}^{j} \end{array}\right].$$

Once a join point ($c_{in}$) is chosen, this also determines the exit point ($c_{out}$) as one of the other corners. The corner designated as the exit point depends on if the number of sweeps is odd or even (Equation (24)). On an even number of turns, the end corner will be on the same side as the start corner; on an odd number of turns, the end is on the opposite side:

(24) $$\left\{\begin{array}{cc} \mathrm{if}\;n_{turns}\;\mathrm{is}\;\mathrm{odd} & c_{1}\to c_{4},c_{2}\to c_{3},c_{3}\to c_{2},c_{4}\to c_{1},\\ \mathrm{if}\;n_{turns}\;\mathrm{is}\;\mathrm{even} & c_{1}\to c_{2},c_{2}\to c_{1},c_{3}\to c_{4},c_{4}\to c_{3}. \end{array}\right.$$

In order to find the fastest traversal path, time costs associated with each edge need to be calculated. The fastest route between start node and the finishing node end needs to be found. These both represent the launch location where the aircraft will start and finish its path from. The edge between the start and the first cell, $E(start,c_{in})$, and the edge between the last cell and the end, $E(c_{out},start)$, are weighted according to the straight line flight time from one to the other in wind presented in Equation (25). All the intermediary edge weights will be the time taken to transfer between cells as well as the survey time for the cell in question for a particular $c_{n}$. These are all shown in Equation (26):

(25) $$time\left(start,c_{in}\right)=\left(\frac{∥\left[\begin{array}{c} x_{start}\\ y_{start} \end{array}\right]-\left[\begin{array}{c} x_{c_{in}}\\ y_{c_{in}} \end{array}\right]∥}{V\cos\left(\arcsin\left(\beta \sin\left(\arctan\frac{x_{start}-x_{c_{in}}}{y_{start}-y_{c_{in}}}\right)-\psi_{w}\right)\right)+V_{w}\cos(\arctan\frac{x_{start}-x_{c_{in}}}{y_{start}-y_{c_{in}}})-\psi_{w}}\right),$$

where [$x_{start},y_{start}$] are the coordinates for $c_{in}$ and [$x_{end},y_{end}$] are for $c_{out}$.

(26) $$d_{p,k}=\left\{\begin{array}{cc} time\left(c_{out}^{j},c_{in}^{j+1}\right)+t_{conv}^{c_{in}}, & p>1\&p<n_{cells},\\ time(start,c_{in}), & p=1,\\ time(c_{out},end), & p=n_{cells}, \end{array}\right.$$

where *p* is the index for the column of nodes.

**Figure 16:**
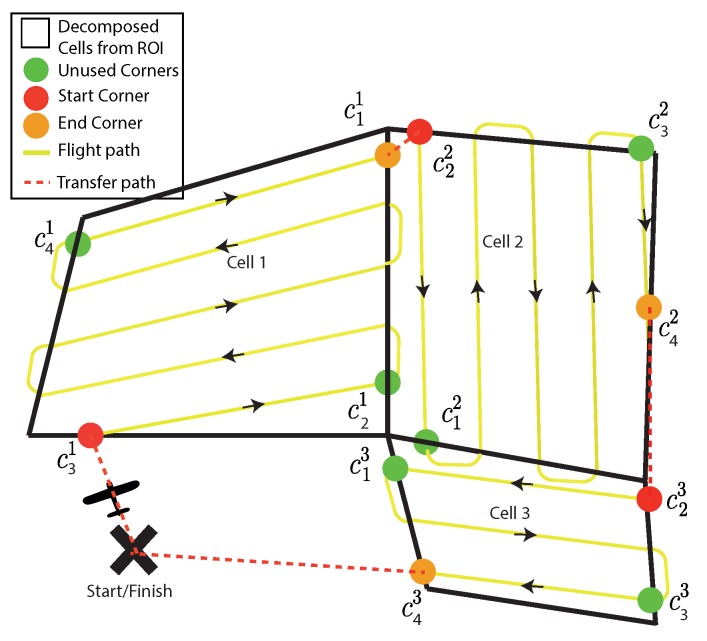
Example traversal of a final decomposition, where the fastest route in wind is $Start\to c_{3}^{1}\to c_{1}^{1}\to c_{2}^{2}\to c_{4}^{2}\to c_{2}^{3}\to c_{4}^{3}\to end$.

**Figure 17:**
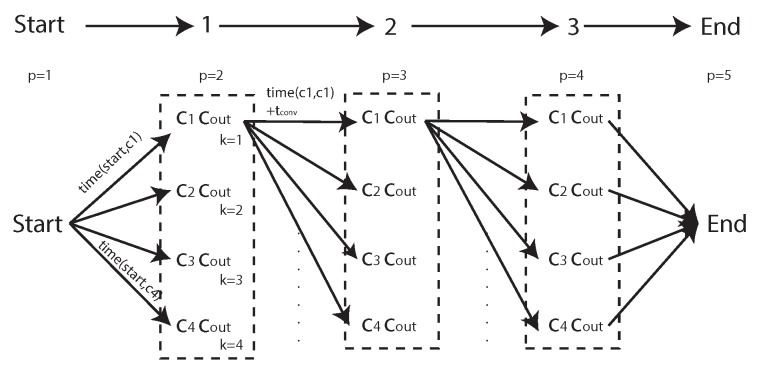
Above: the cell connectivity graph formed from cell adjacency only; Below: cell connectivity graph is expanded to give the full traversal graph including all corner combinations.

Using the simple three cell decomposition seen in Figure 16, the directed graph generation can be outlined. An initial cell connectivity graph is used to generate the initial cell traversal order, seen at the top of Figure 17.

The aircraft is forced to start the survey at the closest cell from its take off location, and the aircraft can only fly between adjacent cells. These simplifications greatly reduce the search space; while this is not optimal due to the rules used, it would be close. As this is an NP-hard problem, for a more complex ROI with more decomposed cells, finding an optimal path would take a prohibitively long to calculate.

The cell connectivity graph is expanded to include all the corner combinations seen at the bottom of Figure 17. By assigning weights to each edge according to Equation (26), the shortest route can be found from “start” to “end”. The shortest path can be found with one of the many standard algorithms. In this case, Dijkstra’s algorithm is used.

## 6. Results and Discussion

In order to assess the improvements offered by the proposed algorithm, paths and flight times will be generated and calculated for a number of example polygons; the first of these is based on a real field in the UK found using Google Earth. In addition to the proposed algorithm (FTIW), other techniques from previous literature (NT [25] and MSA [28]) will also be tested for comparative purposes. Using a real field, it will be shown why accounting for the wind is important, and how the optional cells can make the shape of the decomposed polygon easier and faster to traverse. In addition to comparison using a real field, random polygons are generated in a Monte Carlo simulation in order to calculate an average performance gain figure.

### 6.1. Examples

**Figure 18:**
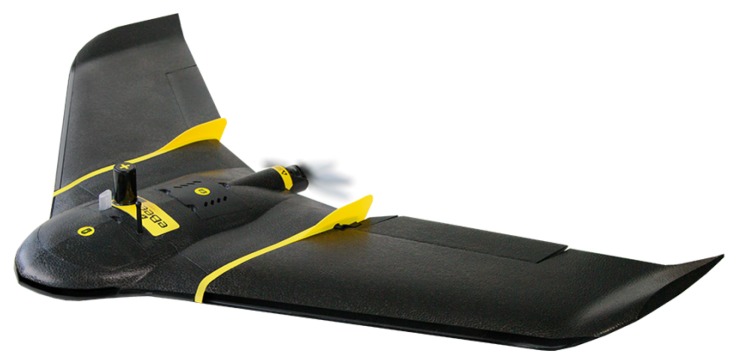
eBee example survey aircraft [38].

The following examples will be simulated using a model of a typical slow flying survey UAV, similar to the senseFly eBee (Figure 18). The survey parameters are chosen based on the requirements for the survey. In this simulation, an orthophoto is required, which only requires a low percentage side/overlap of 40% in the visual spectrum. There was a requirement for a GSD of 0.021 $\frac{m}{pix}$, making the required height to fly approximately 100 m. With the required overlap/sidelap of 40% ($w_{s}=\;0.4,w_{o}=\;0.4$), the distance between tracks is 88 m. The turn rate of the aircraft ($\dot{\psi }$) and its airspeed define the radius of the turn manoeuvres based on $R=V/\dot{\psi }$. The wind is from the north with a speed of 5 m/s. Note that while in general aviation this is not considered particularly strong, this represents approximately half the airspeed of the survey aircraft. The full set of flight parameters is given below:

(27) $$\begin{array}{ccccc} \psi_{w}=180^{\circ } & V_{w}=5m/s & V=10m/s & \dot{\psi }=0.7rad/s & D_{x}=88m,\\ w_{s}=0.4 & w_{o}=0.4 & GSD=0.021\frac{m}{pix} & h=100m. \end{array}$$

**Figure 19:**
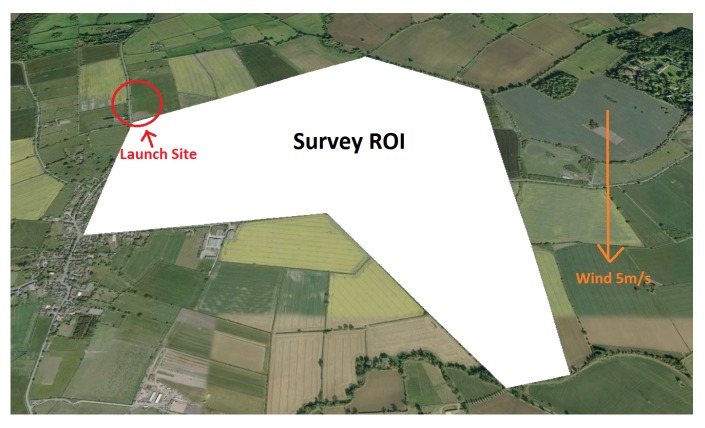
Large example farm field to survey, with a northerly wind of 5 m/s, also showing a launch and landing point for the survey aircraft.

The first example polygon shown in Figure 19 is an area of 173 Hectares, with a perimeter of 5.9 km in length. The aircraft is launched just off the North West (NW) side of the ROI from a field adjacent to a road for easy access. This location is set to (0,0)m and its known latitude and longitude is used to geo-reference the generated path, which is in local Euclidean co-ordinates. The start position location only affects the cell traversal but needs to be considered, as flying to and from a launch point is a fundamental part of the flight that needs to be accounted for. With all parameters defined, a path is generated using the proposed technique for the new cost function and optional cells, and two further paths are generated using NT and MSA, with no optional cells. For each polygon, the algorithm is ran at 40 different polygon rotation angles between $0^{\circ }$ and 180${}^{\circ }$. The overall flight times will be calculated using the survey model defined in Section 5.

**Figure 20:**
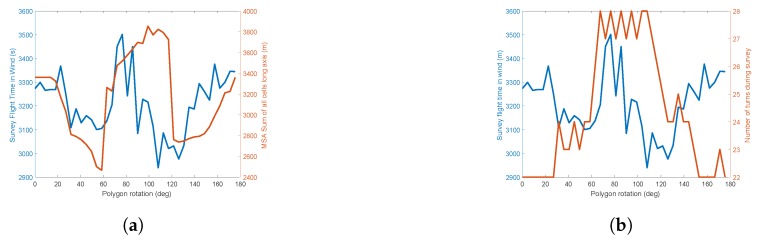
Cost function outputs across a range of polygon rotation angles, comparing cost functions FTIW to both MSA and NT. (**a**) Flight time in wind vs. MSA; (**b**) flight time in wind vs. number of turns.

Figure 20 shows the raw output costs of the flights by angle of rotation, as determined by each of the respective cost functions. As mentioned, the polygon rotation is chosen based on the minimum cost from the cost function. Figure 20a compares FTIW to the MSA cost function. All three cost functions have different outputs: MSA outputs metres, FTIW outputs seconds and NT outputs number of turns. This means that they cannot yet be directly compared. To this end, the paths generated by minimising MSA and NT cost functions will have their flight times calculated by the FTIW model. This means a direct time comparison between them all can be performed. It is clear, however, that they both respond differently, accounting for different factors. The optimal rotation from MSA is $58.5^{\circ }$, whereas, for FTIW, the rotation was 108${}^{\circ }$. In Figure 20b, the NT cost function has multiple winning rotations with the same number of turns showing that the lower resolution of output offered by this function is a disadvantage. It can also be seen that the lowest cost FTIW angle represents one of the highest costs for the NT cost function.

**Figure 21:**
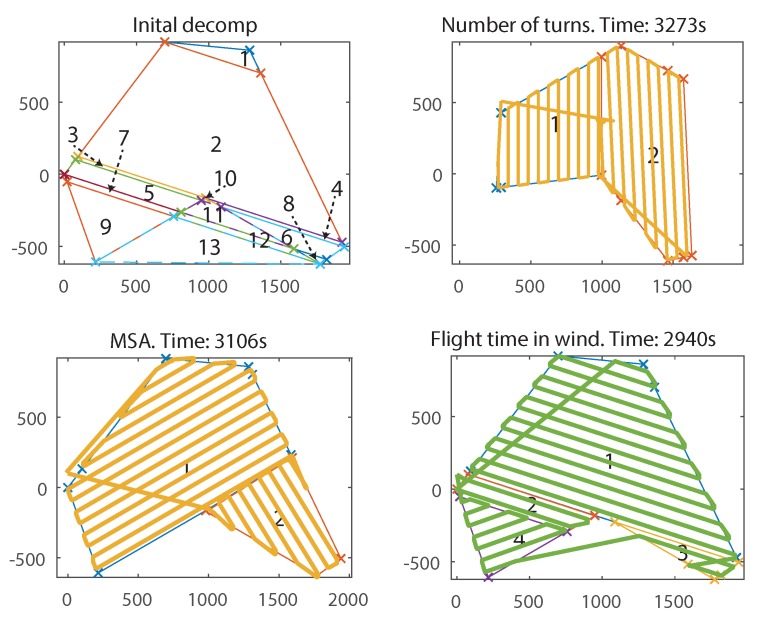
(**Top Left**) Initial decomposition of the example survey field in from Figure 19 at the best polygon rotation angle; (**Top Right**) decomposition and path generated from NT cost function. (**Bottom Left**) MSA cost function; (**Bottom Right**) fastest survey path, FTIW cost function

The final decompositions and paths are shown in Figure 21. It can be seen that the flight time for NT was 3273 s, and MSA was 3106 s, whereas FTIW took just 2940 s, representing an improvement of 10% and 5%, respectively. Closer examination of the results in Figure 21 (BR) reveals that cells 1, 2, 3, 4, and *optional* cell 10 are combined to make the largest convex polygon possible with a long axis, and thus the survey sweep direction mostly perpendicular to the wind. As a result, the FTIW solution has four cells, instead of the two from MSA and NT. This would, at first glance, seem less efficient due to the wasted time in transit between them; however, the algorithm has found that, due to the high relative wind strength, flying perpendicular to the wind outweighs the extra time from having to transfer between more cells.

**Figure 22:**
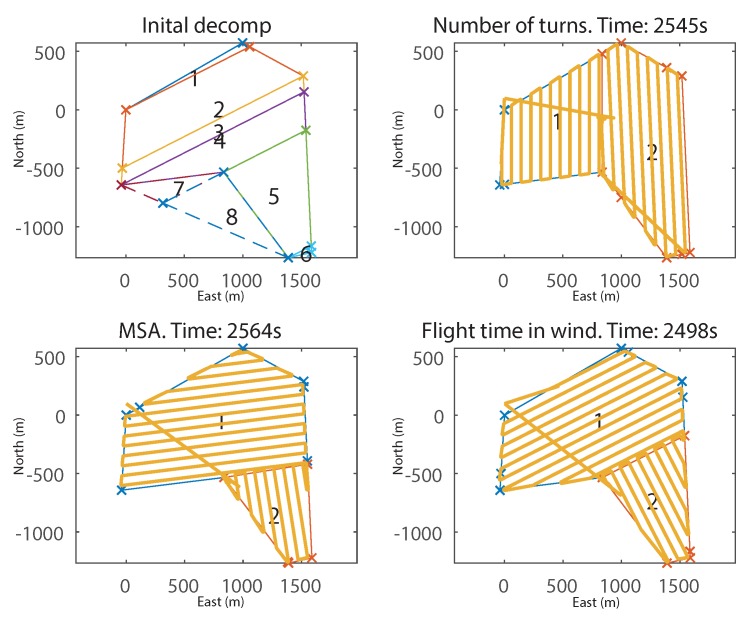
Final decompositions and flight paths generated from the example survey field in Figure 19 in zero wind, for FTIW, MSA and NT.

To demonstrate the importance of accounting for wind, the wind strength was set to zero and the same survey was re-generated. The decompositions and paths are shown in Figure 22. It can be seen that while the proposed method has still found a faster solution than the others, the improvement is greatly reduced: 1.8% for NT and 2.6% for MSA. They have all produced decompositions of two cells, and FTIW made no particular attempt to align the cells in any particular direction (because there is no favourable direction due to the lack of wind); however, minor improvements are still made due to the inclusion of external cells.

**Figure 23:**
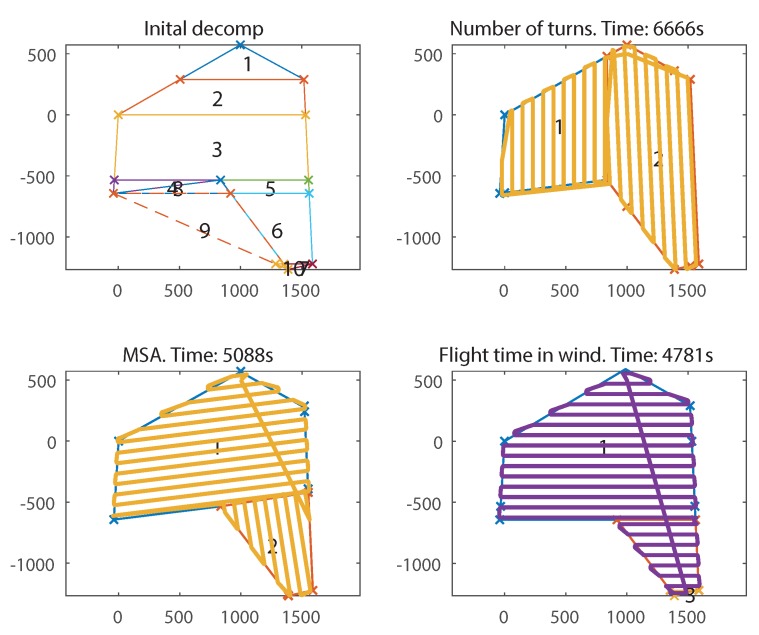
Final decompositions and flight paths generated from the example survey field in Figure 19 in a strong northerly 8 m/s wind, for FTIW, MSA and NT.

To further illustrate the effect of wind, the above simulation was re-run at an increased wind-speed of 8 m/s, which represents 80% of the aircraft’s airspeed. The results can be seen in Figure 23. Because only the wind has changed, the solutions from NT and MSA are identical; however, in this much stronger wind, the flight time has more than doubled. Now the wind is so strong that the advantage of flying perpendicular to the wind has increased significantly. Therefore, the FTIW solution has also used optional cell 8 to create two cells that are both perfectly perpendicular to the wind. This gives a huge 39.4% decrease in flight time compared to NT where both cells have their long axis facing into the wind. There was a much smaller 6.4% improvement over MSA because its decomposition is already, incidentally, more aligned perpendicular to the wind.

### 6.2. Monte Carlo Simulation

**Figure 24:**
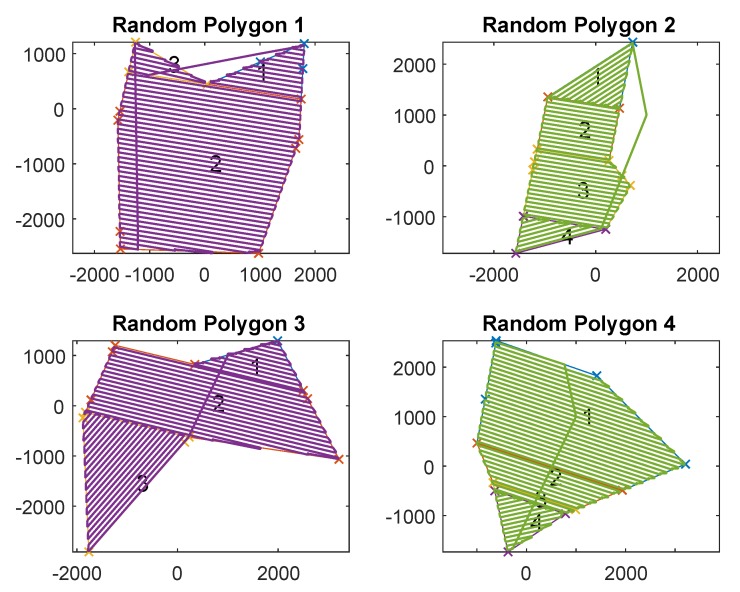
A sample of randomly generated polygons, their optimal decomposition and the associated FTIW path used in the Monte Carlo simulations.

In the previous subsection, a real field was chosen to give a clear demonstration of the different features of the algorithm. However, to give a more balanced assessment, in this subsection, random polygons are generated and the CPP problem is solved for each. A few example polygons and paths from some of the simulation runs are shown in Figure 24. Once again, the proposed technique is compared to previous methods, and a clear improvement is shown. The base parameters of the Monte Carlo simulation are six or seven vertex polygons with an average radius of 2 Km and a wind speed of 5 m/s. However, additional simulations have been performed with modified parameters (shown in Table 2) to assess the effect of the proposed algorithm under a variety of conditions.

Table 2 shows the average improvement across multiple polygons of FTIW over NT and MSA. From these Monte Carlo results, it is clear that this algorithm offers a quantifiable advantage over previous methods. The overall average percentage improvement from the 50 polygons with 5 m/s wind-speed is 7.95% for NT and 9.02% for MSA.

Two simulation runs have been used to further illustrate the importance of accounting for wind. One run was performed at a wind-speed of zero (row 3) and the other was at 8 m/s (row 4), which is a β of 0% and 80%, respectively. With no wind, the proposed algorithm performed slightly better at 1.3% and 1.7% improvement, whereas, at 8 m/s, it considerably outperformed the previous methods at 26.7% and 30%.

The irregularity of the random polygon generation was increased for one run of 10 polygons (row 6). By increasing the standard deviation of each vertice’s random distribution to the polygon centre from 50% average radius to 80%, we can observe how effective the algorithm deals with more irregular (“spiky”) polygons. As can be seen in Table 2, the average improvement is increased to 8.9% and 10.6% for NT and MSA, respectively. This improvement is attributed to the inclusion of optional external cells, which can be used to “clean-up” deep notches in the ROI.

Simulation runs 2 and 5 are to show that polygons with less vertices and smaller random polygons still had comparable results. They do have comparable results where the simulation run of 10 polygons with an average radius of 1km, did well with a 9.4% and 12.5% improvement over NT, and MSA, respectively. A more simple 20 polygon run using six vertex polygons performed similarly well at 8.1% and 7.6%.

## 7. Conclusions

This paper proposes a novel CPP method, designed specifically for low-speed fixed-wing aircraft, such as precision agriculture survey UAVs. The method is developed to optimise a path for covering a polygonal ROI, and as part of this, a calculation for the time taken to transverse a convex polygon in wind is developed. It is shown that wind has a huge effect on survey time and it has been proved mathematically that, in uniform wind, flying perpendicular to the wind confers a flight time advantage over surveying parallel to the wind.

With known aircraft/survey parameters, a complex concave ROI polygon can be optimally decomposed into smaller convex polygons. This is achieved using a “bottom up” dynamic programming approach, which finds the time-optimal convex decomposition within its search space. Taking advantage of the features of a fixed wing UAV, the novel addition of external cells that can be optionally induced during the DP recombination phase gives the CPP the option of overflying corners, which, in turn, may lead to decreased overall flight time. Finally, a full flight path is generated that traverses all the decomposed cells.

To demonstrate clear improvement over previous CPP methods, a Monte Carlo simulation was run where coverage paths were generated for a large number of randomised polygons. A significant improvement was shown over previous methods for typical survey conditions of between 8% and 9%, demonstrating consistent robust performance.

It was also shown that the advantages offered by this method varies greatly due to the type of polygon, wind strength and direction. In particular, wind is shown to have such a large influence that, in one example in Section 6.1, an improvement of 40% was shown in flight time in high wind. This is a huge improvement, yet, at first glance, might seem unrealistic. However, these small aircraft fly at low airspeeds which sometimes can be close to the wind-speed. In these high wind-speed situations, this work has shown that the wind can have a significant effect on the CPP. It is therefore essential that this technique is used, otherwise considerable time could be wasted flying directly into wind. In particular, polygons with notches (or other small features that increase the complexity of the polygon) and polygons with their long axis being close to perpendicular to the wind direction will see the most benefits for using this method.

In this body of work, only simulation studies have been preformed. As the presented algorithm finds the optimal solution, this results in it being computationally incentive, meaning that it not ideal for use for mission planning in the field. However, work is being conducted to extend the algorithm to a recursive greedy solver, which will give a near optimal solution but in an acceptable time. As this is to be used in the field, extensive flight testing will be conducted during this work. There is a big desire to conduct real world testing, in order to prove the survey model in wind is accurate. As we assume that the wind is steady and uniform, the path following and airspeed tracking of the vehicle control is perfect, and errors as a result of these may accumulate. While these assumptions have been discussed in previous work by this author [29], they need to be tested in future work.

## Figures and Tables

**Table 1:** Dynamic Programing (DP) formulation comparison (number of solutions calculated).

Cells Included	Brute-Force	“Top-Down”	“Bottom-Up”
1,2,3	5	4	1
1,2,3,4	15	8	1
1,2,3,5	15	10	1
1,2,3,4,5	52	17	2
**Total**	**87**	**39**	**5**

**Table 2:** Monte Carlo simulation results, 70 random polygons runs.

		FTIW Improvement over NT		FTIW Improvement over MSA	
#	Simulation Description	Av Time(s)	Av Percentage(%)	Av Time(s)	Av Percentage(%)
1	10 polygons	815 s	5.4%	840 s	5.37%
2	20 polygons 6 vertices	1367 s	8.1%	1174 s	7.6%
3	10 polygons $V_{w}$ = 0	169 s	1.3%	191 s	1.7%
4	10 polygons $V_{w}$ = 8	3417 s	26.67%	4309 s	30.01%
5	10 polygons Av Radius 1Km	655 s	8.9%	792 s	10.6%
6	10 polygons increased irregularity	718 s	9.4%	960 s	12.5%
